# Reconstruction of Lower Eyelid with Nasolabial Flap for Anterior Lamella and Turnover Flap for Posterior Lamella

**DOI:** 10.1055/s-0041-1742177

**Published:** 2022-02-03

**Authors:** Vikas Malviya, Sakshi Goyal, Vishal Bansal

**Affiliations:** 1 Department of General Surgery, L. N. Medical College and J. K. Hospital, Bhopal, Madhya Pradesh, India

**Keywords:** eyelid defects, nasolabial flap, turnover flap

## Abstract

Reconstruction of full-thickness eyelid defects is done to provide a mobile lid with corneal protection, having good aesthetic quality, and acceptable donor site morbidity. Various flap procedures have been described and used for the lower eyelid reconstruction; however, the nasolabial flap is rarely employed. It is a random pattern cutaneous flap with redundant blood supply from the perforating branches of the facial and angular arteries and can be used as an inferiorly or superiorly based flap. Here, we aim to present the clinical results of using the superiorly based nasolabial island flap for reconstruction of anterior lamella and turnover/hinge flap of infraorbital skin and palpebral conjunctiva with support of conchal cartilage for reconstruction of posterior lamella for lower eyelid defect. To our best knowledge, this reconstructive combination of flaps has not been described previously for total and full-thickness posttraumatic defect of lower eyelid.

Reconstruction of the eyelids is a challenging task for plastic and reconstructive surgeons and is mostly performed due to trauma, tumor resection, or less commonly, congenital abnormalities. 1 2 Reconstruction of full-thickness defect of eyelid needs proper preoperative planning and selection of appropriate flap coverage for good functional and aesthetic outcome.

Lower eyelid defect are less complex than upper eyelid as the lower eyelid is less mobile, shorter in height with minimal contribution to lid closure. The aim of lower eyelid reconstruction is to provide a functional lid with mobility and globe protection with preservation of lacrimal duct function. The other goals are good aesthetic result and minimal donor site morbidity. The selection of appropriate reconstructive option depends on assessment of the eyelid defect in terms of its location, size, extent, and thickness of the defect.

Full-thickness defect of lower eyelid needs reconstruction of anterior lamella and posterior lamella. There are various flap alternatives for anterior and posterior lamella defect of lower eyelid. In this case report, we described the use of nasolabial flap for reconstruction of anterior lamella and turnover/hinge flap of infraorbital skin and palpebral conjunctiva with support of conchal cartilage for reconstruction of posterior lamella for lower eyelid defect. This reconstructive combination of flaps has not been reported previously for total and full-thickness posttraumatic defect of lower eyelid.

## Case History

A 60-year-old male patient presented with history of road traffic accident 3 years back with complaint of posttraumatic left lower eyelid defect associated with incomplete closure of the eyelid, excessive lacrimation, redness, and burning with irritation of left eye.

**Fig. 1:**
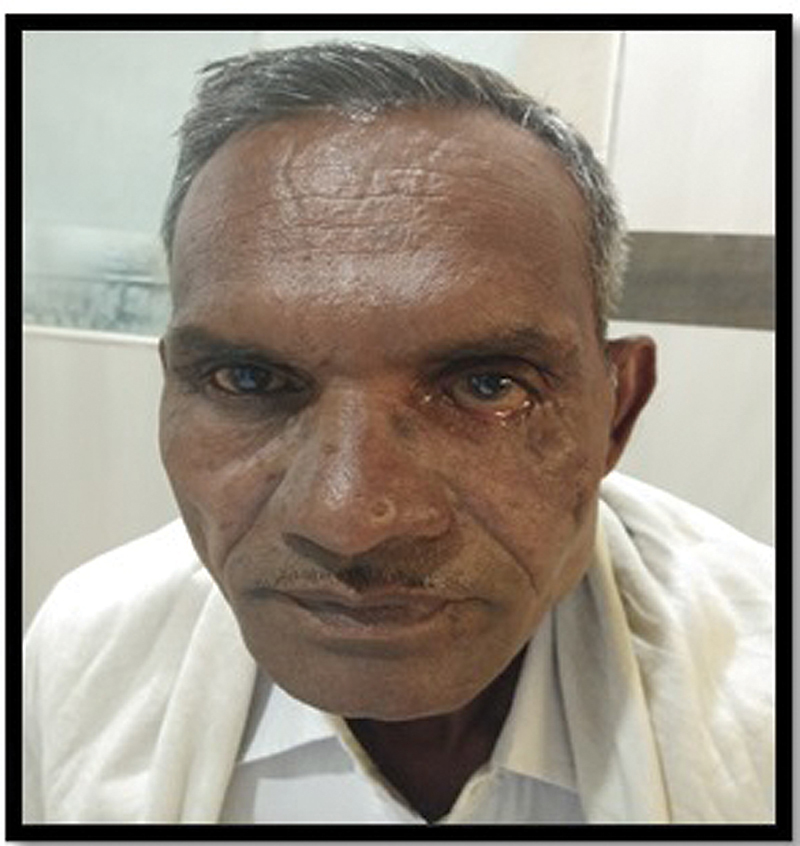
Clinical picture showing posttraumatic left lower eyelid defect.

Clinical examination revealed complete loss of the skin, subcutaneous tissue, orbicularis oculi muscle, the tarsal plate with exposed palpebral conjunctiva of left lower eyelid. Other structures of eye like cornea, sclera, bulbar conjunctiva, lower fornix, and lacrimal apparatus were intact. There was conjunctivitis and congestion over left eye due to persistent exposure. Upper eyelid was normal in terms of function and cosmesis. There was minimal scarring over the defect site in the infraorbital region ( Fig. 1 ).

## Operative Procedure

**Fig. 2:**
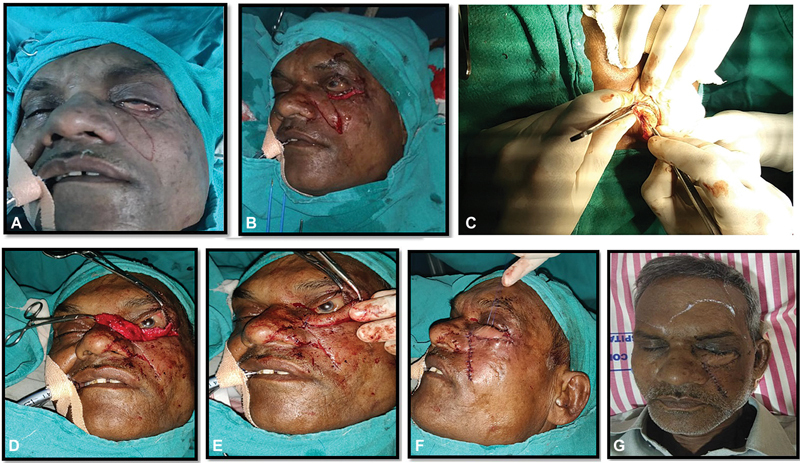
( **A** ) A 60-year-old male patient with left-sided posttraumatic lower eyelid defect. ( **B** ) Lower eyelid defect located at zones 2. ( **C** ) Conchal cartilage harvesting. ( **D** ) Raised superiorly based nasolabial island flap. ( **E** ) Posterior lamellar reconstruction by conchal cartilage graft. ( **F** ) Immediate postoperative view. ( **G** ) 3rd postoperative day view of the patient. 10

Procedure was performed under general anesthesia. First, turnover flap of infraorbital skin was marked and incision made to elevate the flap ( Fig. 2A ). Preexisting exposed palpebral conjunctiva (3–4 mm width) and skin (3–4 mm width) were dissected to harvest the turnover flap of width 6 to 7 mm and length of approximately 3.8 cm ( Fig. 2B ).

For harvesting the conchal cartilage, the patient's left ear was exposed. One percent lidocaine with 1:100,000 epinephrine was infiltrated into the subcutaneous plane on both anterior and posterior surface of the conchal bowl. Incision of 4-cm length was made in retroauricular folds. Soft tissue and muscle layer were incised. Sharp dissection was done to expose the perichondrium over posterior aspect. Conchal cartilage strip of 3.8 cm × 0.6 cm was marked and incision made over the perichondrium. Dissection was done in anterior subcutaneous plane keeping the skin intact and cartilage strip was dissected off taken out as a free graft. After harvesting the conchal cartilage strip hemostasis was achieved with cautery. Skin was closed with ethylone 4.0 suture. A bolster was placed in the conchal bowl for prevention of hematoma.

This strip of conchal cartilage was then inset over the turnover flap of the skin and conjunctiva for reconstruction of posterior lamellae. Conchal cartilage was anchored to the lateral canthal tendon laterally and remnant of tarsal plate medially to serve as tarsal plate and to give support to the reconstructing eyelid ( Fig. 2C ).

The anterior lamella was then reconstructed by using nasolabial flap ( Fig. 2D ). For this, superiorly based nasolabial flap was designed over the left nasolabial region after planning in the reverse keeping the length-to-width ratio 4:1 with 6 cm × 1.5 cm in width.

The dissection of the flap was started caudally in the subcutaneous plane and carried toward the pivot point located at the medial canthal region on a subcutaneous pedicle. This harvested nasolabial flap was transposed horizontally to cover the complete lower eyelid defect over the reconstructed posterior lamella of skin and conjunctiva turnover flap. Donor site of the flap was closed first in two layers after mobilizing both margins. Superior flap margin was sutured to margin of the harvested skin turnover flap medially to laterally. Inferior margin of the nasolabial flap was sutured to the inferior margin of the donor area of turnover flap in two layers ( Fig. 2E ). Finally, on postoperative day 3 the first dressing was removed ( Fig. 2F ).

## Result

**Fig. 3:**
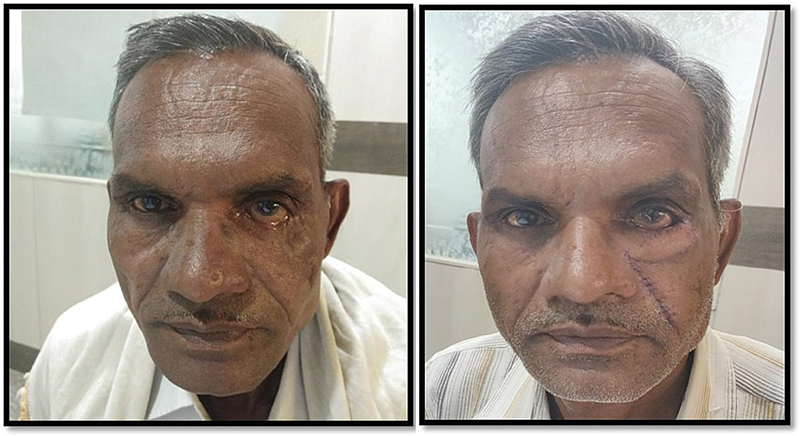
Pre- and postoperative picture of patient.

Postoperative period was uneventful with complete recovery without any complication. Postoperative complications like flap necrosis, wound dehiscence, wound infection, xerophthalmia, entropion, donor site morbidity, or graft failure were not observed. Patient was discharged with skin suture removal at day 7. In the follow-up, patient had no complaints of redness, irritation, excessive watering, or tearing of the left eye. On examination, there were no signs of epiphora, corneal ulceration, and congestion of conjunctiva. Patient was satisfied and had a complete occlusion of left eyelid to cover the globe ( Fig. 3 ).

## Discussion

The functions of eyelid are protection of the globe from injury, regulation of light, moisturizing the eye by making tear film, and pumping the excess tears to the conjunctival and lacrimal sac. Eyelid defects after trauma and oncological excision may impair these important functions of eyelid. Reconstruction of posttraumatic defects of lower eyelid is a challenging task for plastic surgeons which needs proper preoperative planning and execution of meticulous technique.

For lower eyelid defects, each layers of lower eyelid must be reconstructed for better aesthetic and functional outcome. These layers include the posterior lamella, consisting of the conjunctiva and the tarsal plate, and the anterior lamella, consisting of the pretarsal orbicularis oculi muscle and the lower eyelid skin. 3

Reconstruction of lower eyelid defect starts with ophthalmologic examination and includes thorough analysis of the defect in terms of location and layers involved. Spinelli and Jelks divided the periorbital region into five zones—zone 1, the upper eyelid; zone 2, the lower eyelid; zone 3, the medial canthus; zone 4, the lateral canthus; and zone 5, the surrounding tissues 4 ( Fig. 1 ). As per location, the defects of lower eyelid are included in zone 2.

Full-thickness eyelid defects require reconstruction of both the anterior and posterior lamellae. Both lamellae cannot be grafts since at least one of them must have a blood supply. The correct options include: (1) anterior graft + posterior flap; OR (2) anterior flap + posterior graft; OR (3) anterior + posterior flap. 5

For superficial defects involving up to 20% of lower lid length, primary closure is usually possible in older patients. Similarly, small full-thickness lower lid defects are closed primarily. 6 When primary closure is not feasible, various flap alternatives developed with the aims of functional restoration and aesthetic improvement of the lower eyelid zones can be employed, such as the semicircle (Tenzel) flap, superiorly based tarsoconjunctival advancement (Hughes) flap, upper eyelid myocutaneous (Tripier) flap, transposed cheek (McGregor) flap, cheek rotation and advancement (Mustarde) flap, and supraorbital (Fricke) flap. 7 8 9 Various autologous tissue grafts are also used for posterior lamella reconstruction of eyelid in case of full-thickness defect. Autologous tissue grafts are mostly used to resurface the defects of the posterior lamella, this can be accomplished with a tarsoconjunctival graft, but buccal or hard palate mucosal grafts are better options. 10

In the present case report, for posterior lamella reconstruction hinged/turnover flap of skin and conjunctiva are used with support of conchal cartilage graft. Skin of width 2 mm was marked from the margin of lower palpebral conjunctiva and elevated after making incision with mobilization of palpebral conjunctiva meticulously. In literature, most turnover flaps are currently being used in the lower extremities. Other sites where it is being used for reconstruction is face especially for nose as nasal lining. Turnover flaps are often utilized as alternatives to more traditional flaps, especially in situations where traditional flap viability is limited. 11

Hinged/turnover flaps are intended for the coverage of through-and-through defects, where transposition of the flap no longer takes place at skin surface level, but rather the flap is turned over 180 degrees around an axis that lies level with the skin. The hinge flap possesses a unique motion whereby a flap of tissue folds over on its pedicle like a page of a book to fill the depth of a defect. After the epithelial free hinge flap is inset to fill the wound's base, cutaneous coverage is provided by a separate graft or flap. The use of the hinge flap restores the natural contour by introducing a volume of tissue for repair that a graft alone or a single flap cannot provide. 12

The nasolabial flap is a useful cutaneous pedicle flap with versatile and robust blood supply and used for anterior lamella reconstruction. Along with its common use for midfacial defects, the use of the nasolabial flap for the reconstruction of the lower eyelids has various advantages: (1) ease of dissection of the flap, (2) ease of access of the flap to zones 2 to 5, (3) skin color and texture matches with remaining eyelid tissue, (4) provision of eyelid–cheek transition according to aesthetic norms, and (5) minimal donor site morbidity, as the donor site scar is hidden within the nasolabial fold. 13 14

## Conclusion

The superiorly based nasolabial island flap provides a reliable means of obtaining good wound healing with acceptable aesthetic and functional results of both the donor site and reconstructed area. Reconstruction of the eyelid with mucosal lining, support, and cutaneous cover to achieve normal appearance and function, are still a challenge to the surgeon.
